# Assessment of Language Functions in Patients With Disorders of Consciousness Using an Alternative Communication Tool

**DOI:** 10.3389/fneur.2021.684362

**Published:** 2021-07-20

**Authors:** Katarzyna Kujawa, Grzegorz Zurek, Agnieszka Kwiatkowska, Roman Olejniczak, Alina Żurek

**Affiliations:** ^1^Department of Biostructure University School of Physical Education in Wroclaw, Wroclaw, Poland; ^2^Neurorehabilitation Clinic, Wroclaw, Poland; ^3^Neurologopedic Practice Hear My Voice, Bydgoszcz, Poland; ^4^Institute of Psychology, University of Wroclaw, Wroclaw, Poland

**Keywords:** neurorehabilitation, unresponsive wakefulness syndrome, disorders of consciousness, cognitive functions, eye tracking

## Abstract

This study aimed to describe the percentage of tasks involving language functions that were completed by patients diagnosed with disorders of consciousness, as observed during neurorehabilitation conducted for different periods of time using an alternative communication tool. The project involved six participants, who were observed for 1 month, 6 months, and 1 year. The patients were asked to solve tasks involving language functions with the use of an eye-controlled device. The language functions were evaluated on the basis of the average number of tasks performed by the patients, which was 70.45% for the whole subject group. It is not entirely clear what determined the changes in language functions during the research. It is crucial that patients performed the presented tasks even though their state of consciousness, as confirmed through medical documentation (unresponsive wakefulness syndrome), did not suggest the possibility of establishing any contact with them.

## Introduction

Interpersonal communication is a key element in every person's daily life. The communication of information makes it possible to understand each other's needs; people need to be heard out so they can communicate their thoughts in a way that is available to them. But not every person has the capability to communicate with others verbally due to damage to the speech organs, consequences of diseases, or brain injuries. What can be of considerable assistance in such situations is augmentative and alternative communication (AAC). According to the American Speech–Language–Hearing Association, AAC is a decision-making system that examines individual methods of communication and determines their effectiveness in people with various speech defects. According to the AAC theory, there is a possibility of communicating through gestures, pictographs, or speech generators (1–4). Sometimes, as a result of damage to the central nervous system (CNS), the patient's only way of communicating with other people is through eye movements (5, 6). Depending on its severity, damage to the CNS may result in locked-in syndrome (LIS), a minimally conscious state, a coma, or unresponsive wakefulness syndrome (UWS). Patients in a minimally conscious state (MCS) have retained but minimal consciousness. The state of minimal consciousness is distinguished by the presence of behavioral signs of awareness of oneself, others, and the environment. Patients in this state react to stimuli; for example, we can expect a response to simple verbal commands (“close your eyes,” “look at me”). Patients with locked-in syndrome (LIS) are fully aware of what is going on around them, but they are paralyzed and unable to communicate verbally and through movement; they can only blink their eyes. Comatose patients do not have the capability to communicate with the people around them, lack reflexes, and demonstrate severe disturbances in awareness. UWS involves a lack of response to external stimuli, even though reflexes are present. It is characterized by an unawareness of the surroundings combined with retained wakefulness (7–10). As many as 40% of diagnoses made following an injury to the CNS are incorrect; patients are commonly diagnosed as being in an UWS. Unfortunately, misdiagnosis often leads to inappropriate steps taken in neurorehabilitation (11, 12). Moreover, brain tissue injuries are associated with functional disturbances to the brain functions, leading to major disturbances to the cognitive and communicative functions. Because the thinking process is strictly associated with speech, the use of visual communication channel offers a chance to improve the disturbed functions in the case of such injuries (13).

In Poland, just like in many other countries, the main consciousness assessment procedure is the application of the Glasgow Coma Scale (GCS) (14). However, our literature review shows that the most appropriate scale for assessing the state of consciousness is the Coma Recovery Scale (CRS-R) (15–17). The Polish validation of the CRS-R was not available at the time the present study was planned, which is why the diagnosis of patients in our studies was carried out in accordance with the standards then in force in Poland (18).

The aim of the study was to describe the percentage of tasks involving language functions that were completed by patients diagnosed with disorders of consciousness (DOC), who were observed for 1 month, 6 months, and 1 year during neurorehabilitation conducted using an alternative communication tool. Additionally, we wanted to determine the potential of such patients to undergo neurorehabilitation. The study may contribute to changing the perception of these individuals as unfit for neurorehabilitation.

## Materials and Methods

### Characteristics of the Participants

The study included six patients aged 18–62, whose cases were judged through clinical assessment as UWS caused by sudden circulatory arrest (SCA). None of the patients had undergone any neurorehabilitation by eye tracking. The inclusion criteria for this study were as follows: the patient had been confirmed to be in a state of UWS; they had at least one functioning eyeball; and the caregiver provided consent for the patient to participate in the study. The exclusion criteria were as follows: both eyeballs were defective; the caregiver did not provide consent for participation; and the patient's health significantly deteriorated, thus preventing participation in the examination of language functions. After the SCA, standard medical procedures were performed, including physical examination and computed tomography, which indicated severe, irreversible brain damage. The state of consciousness of the subjects was determined by a doctor based on the GCS, and in each person was ≤ 8 points. Due to the procedures in practice in Poland at that time, the GCS was used to assess the awareness of each participant, which was performed before and after the therapeutic intervention (18). Finally, the study involved 3 men and 3 women, who participated in the project for 1 month (2 people), 6 months (2 people), and 12 months (2 people). The different duration of participation in the project (1, 6, and 12 months) was dictated by our intention to obtain an answer to the following question: Did the time devoted to the training of language functions have an effect on the improvement of these functions. All patients were investigated between 4 and 6 months after the incident. SCA resulted from: hemorrhagic right-side stroke in Patients 2, 4, and 6 (*n* = 3); ischemic left-side stroke in Patient 3 (*n* = 1); a suicide attempt in Patient 1 (*n* = 1); and childbirth in Patient 5 (*n* = 1). The data were collected in 2017–2018 during a neurorehabilitation program undertaken by the subjects. The research was approved by the Institutional Bioethical Committee at the University School of Physical Education in Wroclaw, Poland.

The characteristics of the patients with UWS are presented in Table 1.

**Table 1:** Characteristics of the sample.

	**Characteristics**							
**Subject**	**Age**	**Sex**	**Participation time [months]**	**Reasons for SCA**	**Time [months]**	**GCS -B**	**GCS -A**	**Average of results [%] Standard deviation**
P1	18	F	1	Suicide attempt	4	7	7	75.80 9.121
P2	55	M	1	Hemorrhagic right-side stroke	4	7	7	80.00 12.987
P3	40	M	6	Ischemic left-side stroke	5	6	6	63.43 33.430
P4	62	F	6	Hemorrhagic right-side stroke	6	8	8	75.41 16.109
P5	33	F	12	Childbirth	4	7	7	61.22 27.895
P6	49	M	12	Hemorrhagic right-side stroke	5	6	6	66.83 27.014
All								70.45

*Time, interval of time from the critical event to the beginning of the study; P, patient; F, female; M, male; GCS-B, score before the study; GCS-A, score after the study*.

### Device Description

**Figure 1:**
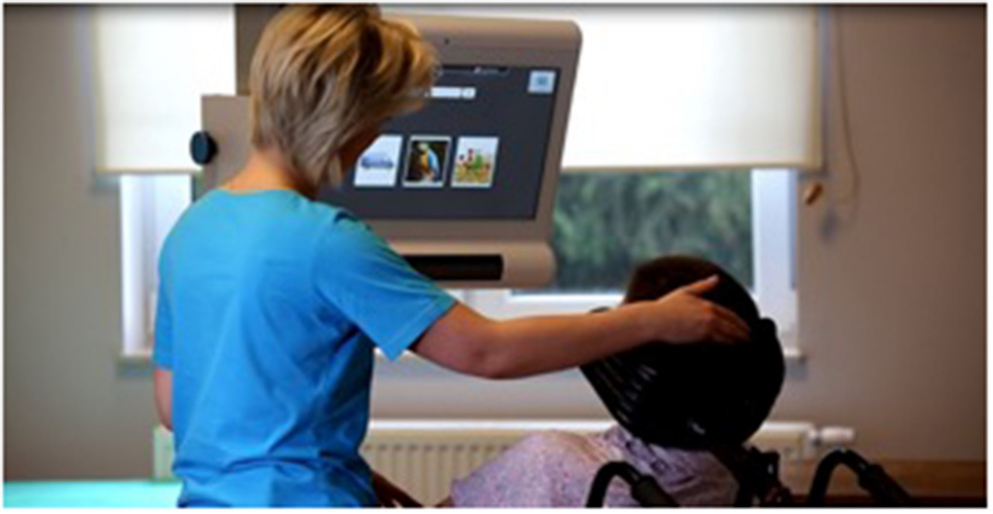
Eye tracker: C-Eye (source: AssisTech).

The collection of data was possible thanks to the use of a device (C-Eye pro) based on eye-tracking (Figure 1). The C-Eye system is based on infrared (IR) illumination, which is invisible and so does not disturb the user. Exploiting IR illumination enhances the effectiveness of the image processing algorithm. The contrast between the pupil and the iris is much higher in the processed frame in comparison to the standard grayscale image. IR generates characteristic corneal reflections called glints. A better contrast and four reflections on the cornea increase the precision when determining the fixation point (19). The device consisted of a monitor mounted on a frame with a metal extension arm, enabling its direct placement in front of the patient's face. The coding device was a computer with specialist software. The system worked based on the patient's eye contact with the device. The patient focused their sight on the relevant element displayed on the screen for a specified amount of time, which allowed the device to read the position of the eyeball(s). After the parameters were initially set up through calibration, it was checked whether the device read its position correctly. Calibration was selected from the device settings menu. In the middle of the screen, a red flashing dot with a white border around it appeared as a point for the patient to look at. The therapist asked the patient to look at the red dot, simultaneously showing its location with the hand. After a few seconds, the dot began to move across the screen. During this time, the therapist verbally and behaviorally encouraged the patient to follow the dot. The system analyzed the patient's sight fixation on the dot and whether they were able to follow it on the screen. Successful calibration was confirmed by the computer displaying a message on the screen that read: “calibration was successfully performed.” After successful calibration, a cursor in the form of a red dot appeared on the screen, which reflected the location of the patient's eye-fixation point. If the patients were not able to fixate their eyes and track the dot, the system announced that the calibration was not successfully performed and thus the patient could not proceed to mark the answers. The device was calibrated before each therapeutic session. More detailed information on the C-Eye software and hardware can be found in earlier publications (20, 21).

### Language Tasks

The tasks for assessing language functions were divided into five categories. The first category included tasks concerning the understanding of single nouns; the second category – understanding single verbs; the third category –understanding the surrounding world; the fourth – understanding sentences; and the fifth – reading whole sentences with understanding.

The tasks from the first and the second category involved selecting one black and white pictogram representing a noun or a verb, respectively, which was read aloud by the device. After hearing the word, the patients were to choose the word they had just heard from among three pictograms (there were always three pictograms to choose from, excluding the category: “understanding the surrounding world”) displayed in the middle of the monitor. The tasks determining the patients' understanding of the surrounding world (Category 3) required a “yes” or “no” answer to the question asked. Understanding of sentence-related tasks (Category 4) consisted of answering the question by matching the appropriate pictogram (from among three available) to that question. The task of reading whole sentences (Category 5) consisted of choosing the sentence heard from among three sentences displayed on the monitor (there were always three sentences available) (Table 2). Each patient had to perform 25 tasks involving language functions during one therapeutic session. They were randomly selected by the software. In order to avoid memorizing the tasks in individual sessions, the patient received a different set of questions.

**Table 2:** Categories (C) with examples of tasks involving language functions.

**Type of task (category)**	**Task read aloud by the device**	**Content of the task displayed on the monitor**	**Correct answer**
Understanding of a single word – nouns (C1)	Point to the picture you will hear called: the thermometer.	Pictograms: •Snail •Thermometer •Robot	Thermometer
Understanding of a single word – verbs (C2)	Point to the picture you will hear called: Dance.	Pictograms: •Pull the sledge •Dance •Cook	Dance
Understanding the surrounding world (C3)	Answer the question: Does the rose have thorns?	The sentence displayed: Does the rose have thorns?Pictograms: •Yes (green pictogram with a tick) •No (red pictogram with an X)	Yes (green pictogram with a tick)
Understanding of sentences (C4)	Answer the question: What became a tourist attraction?	Displayed sentence: The fountain has become a tourist attraction.Pictograms: •Statue •Palace •Fountain	Fountain
Reading sentences with understanding (C5)	Mark the sentence: He was a master of card games.	Sentences: •He was a master of card games. •His eyes were wide open. •I found a big white brick house.	He was a master of card games.

### Evaluation of the Session

Each participant was positioned 50 cm from the screen. The session was conducted by a therapist, a specialist in neurologopedia, and lasted 60 min. It comprised a greeting, the main part – during which the patient performed the tasks of language functions, and a goodbye to the therapist. The breaks during each session were not counted. The frequency of sessions varied due to the patients' changing health condition (colds, doctor's visits, etc.) and lack of willingness to cooperate for unexplained reasons. Willingness to cooperate was assessed once before every session by an experienced neurologopedist by presenting 6 pictograms (“good morning,” “have a nice day,” “goodbye,” “I'm sorry,” “thank you,” “good night”) displayed on the screen. If the patient selected the pictograph “good morning,” the therapist proceeded to practice language functions. In addition, in each session, the first task presented on the monitor was treated as a “test” during which the patient was carefully observed for eye opening and eyeball movements, and thus gaze direction. After the first task was completed, the patient moved on to further tasks, with eye opening and gaze direction monitored throughout. The welcome, the completed “test,” and the observation of the patient with the variables mentioned were treated as conditions for further work, the time for which was limited to no more than 10 min.

During the study, each patient not only learned which pictograms they could use to say “hello” and “goodbye” to their therapist, but also which pictograms to mark in order to ask for a break or finish their session.

### Statistical Analyses

Statistical analyses were performed by calculating mean values (X) and standard deviations (SD) as well as median values (Me) and coefficients of variation (CV). The values for Me and CV were calculated first, separately for N valid outcomes obtained by each participant during the entire intervention, then for N valid outcomes obtained in the entire group during the first and last measurement. A Kruskal-Wallis ANOVA factor analysis was conducted to assess the significance of differences in outcomes, considering separately the N valid sessions of each participant. Finally, the Wilcoxon pairwise ranked order test was used to assess the statistical significance of changes in outcomes across the group in pre- vs. post-intervention measures. The latest version of the *Statistica* package was used (v. 13.1 pl).

## Results

We used the number of tasks completed by a given patient as an indicator of language functions, specifying that 0–5 (0–20%) correctly performed tasks meant a very low percentage; 6–10 (21–40%) tasks performed – a low percentage; 11–15 (41–60%) tasks – a moderate percentage; 16–20 (61–80%) tasks – a high percentage; and 21–25 (81–100%) tasks performed – a very high percentage. The result of 100% indicated that the patient performed all the tasks correctly in a given session.

**Figure 2:**
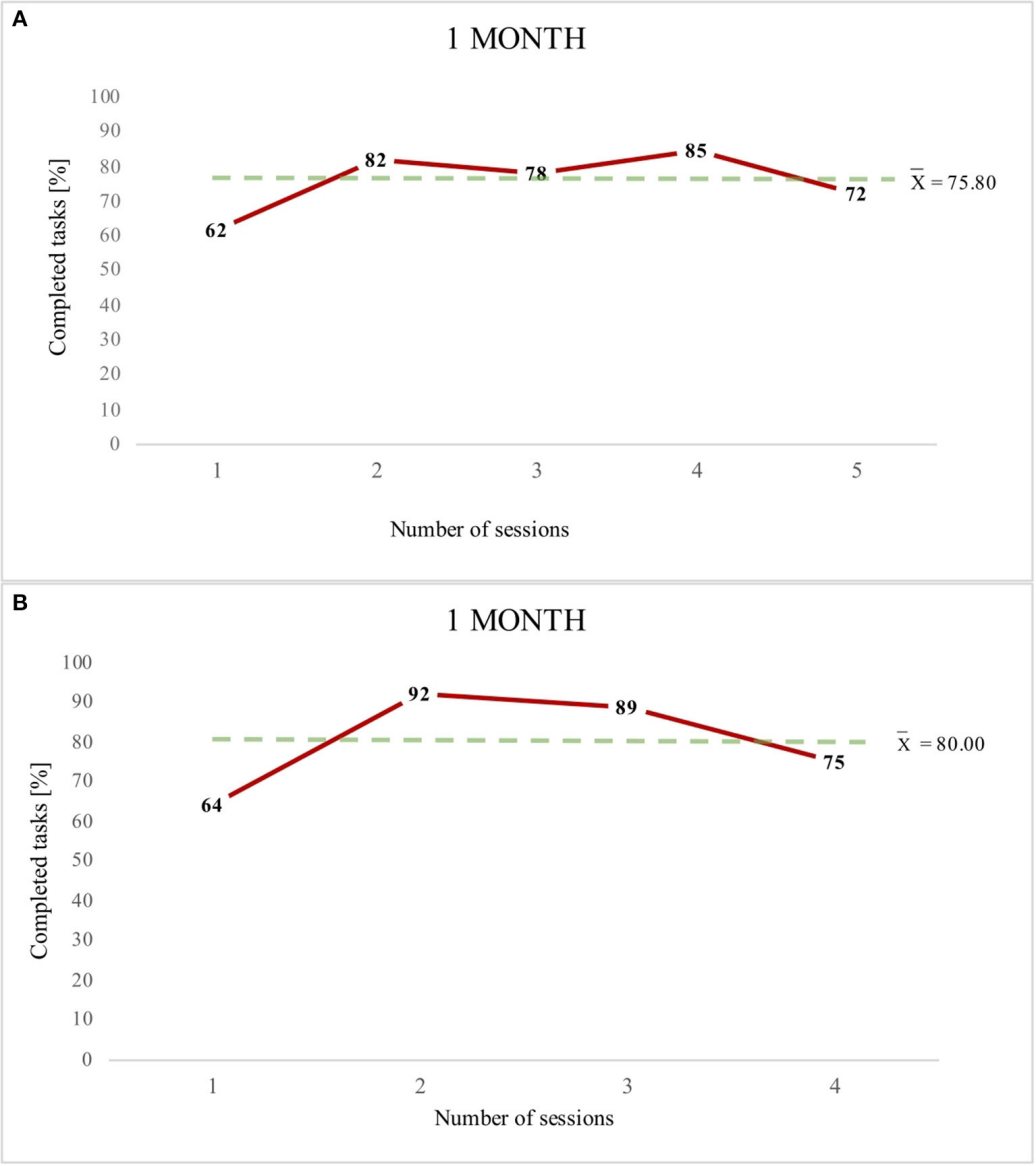
Patients 1- graph **(A)** and Patient 2- graph **(B)** (1 month of work with the device).

**Figure 3:**
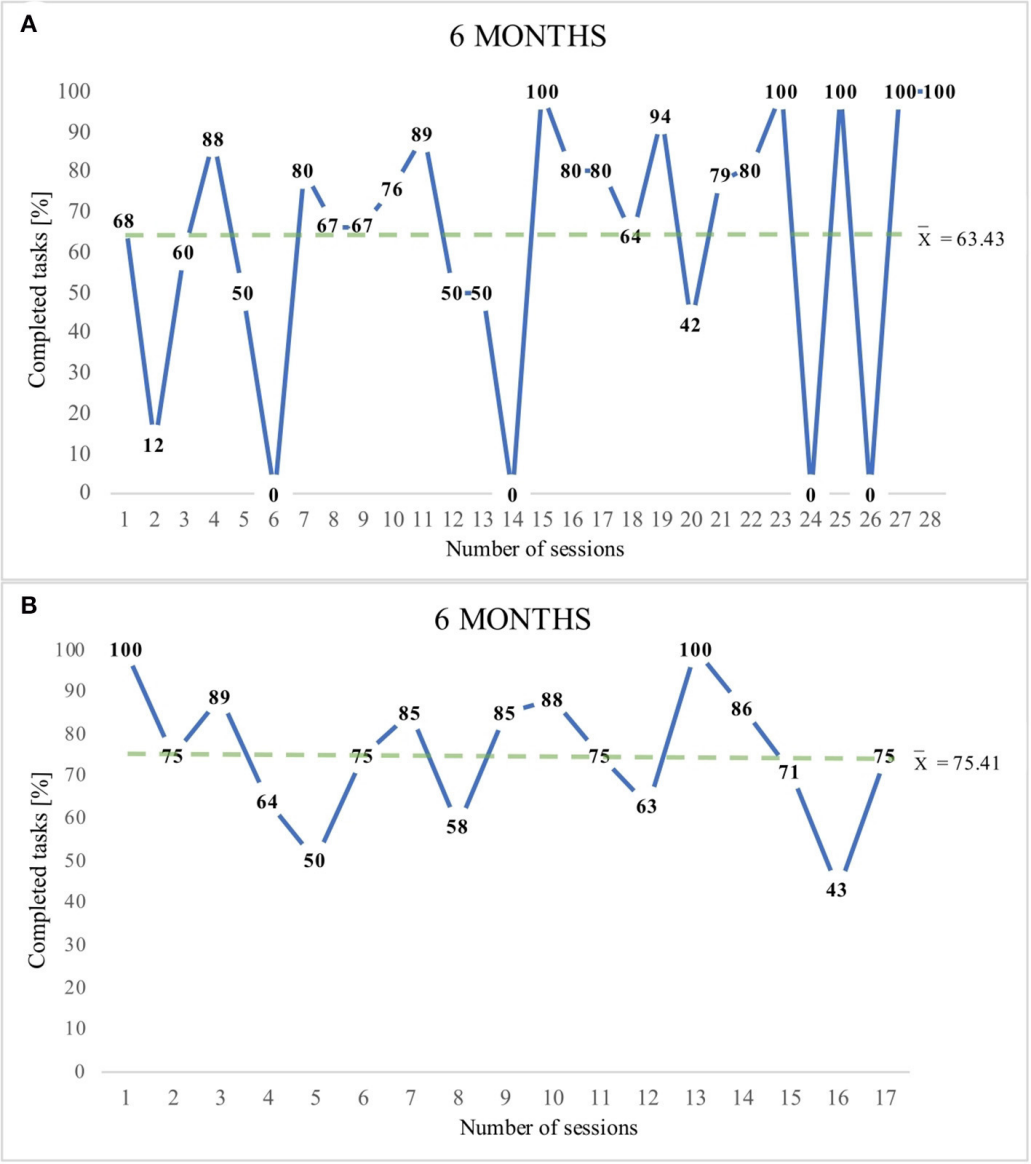
Patient 3- graph **(A)** and Patient 4- graph **(B)** (6 months of work with the device).

**Figure 4:**
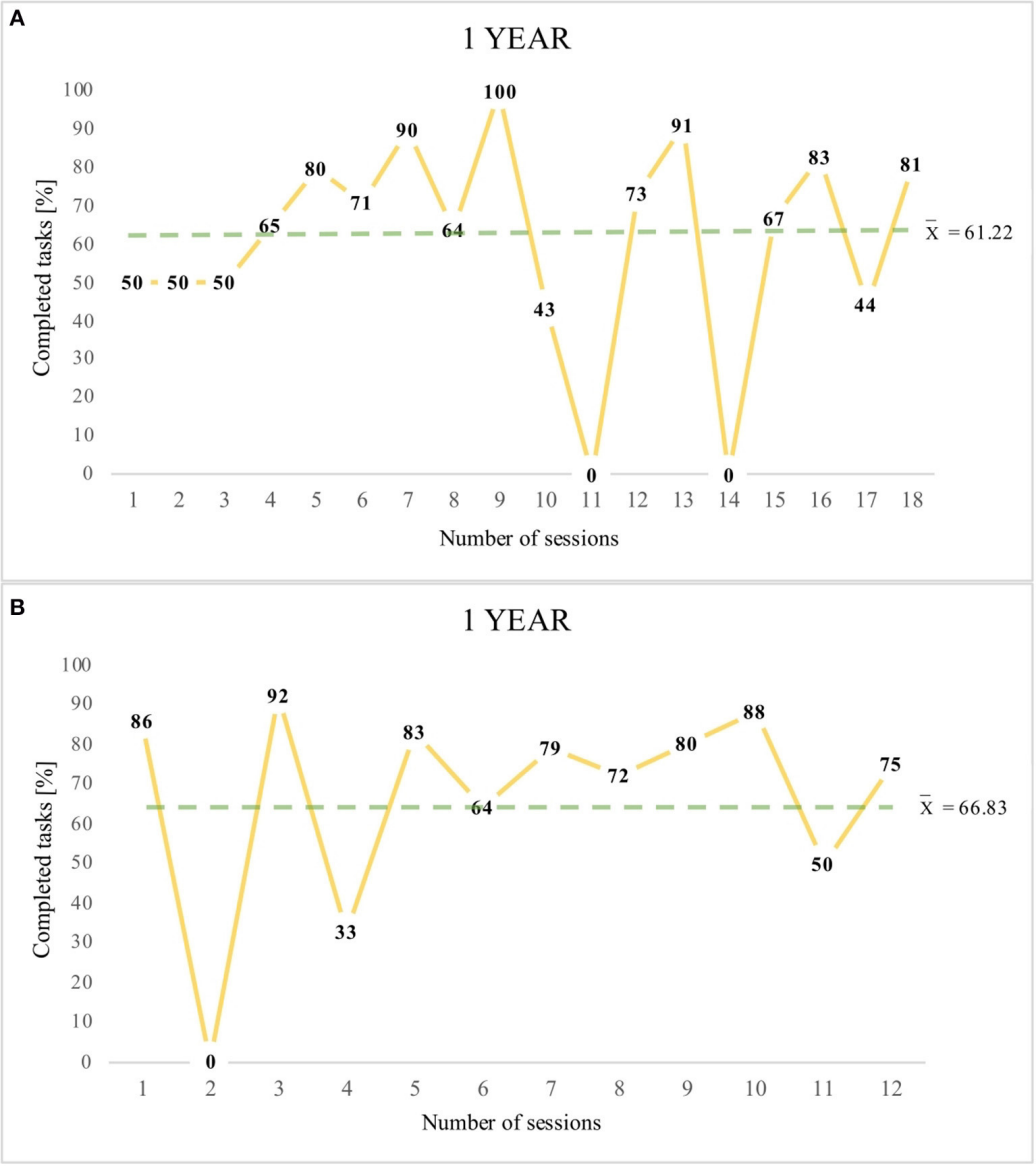
Patient 5- graph **(A)** and Patient 6- graph **(B)** (1 year of work with the device).

Graphs A,B (Figures 2–4) present the percentage results obtained in each therapeutic session conducted using the eye-controlled device. The Y-axis indicates the percentages of the tasks completed. The X-axis indicates the number of sessions, which is the number of times the patient worked with the device. The dotted line indicates the mean result of all sessions for a particular patient. Showing trend lines in the charts was eliminated due to considerable fluctuations in participants' results (0–100% tasks performed).

(6 months of work with the device).

Figure 2 presents the therapeutic sessions of two patients (P1 and P2), which were conducted for 1 month. Despite the small number of sessions, there was an improvement in the results in both participants. They achieved mean results indicating a high percentage of language functions (P1 – 75.80% and P2 – 80% of correctly completed tasks).

Patient number 3 underwent 28 sessions during the 6 months of neurorehabilitation, achieving a high result (73.43%), whereas P4 underwent 17 sessions, also ending up with a high result (75.41%).

Patient number 5 underwent 18 therapeutic sessions and achieved a high result (61.22%), and P6 participated in 12 therapeutic sessions, also achieving a high result (66.83%). The analysis of the study reveals that all participants cooperated and achieved a mean result of 70.45% (a high percentage).

In Patients 3, 5, and 6, in some of the sessions, there was a considerable decrease in the efficiency of task performance, and sometimes, these patients did not cooperate with the therapist at all. According to the report made each time after the session by the neurologopedist, the reasons for this should be seen as stemming from the participants' malaise and lowered basic mood, and sometimes from the atmospheric conditions. In addition, there were intervals between sessions, sometimes even long ones, which were caused by the participant's illness. The study showed no changes in the Glasgow scores before and after the therapeutic intervention. Table 3 shows the results of the statistical analyses.

**Table 3:** Results of statistical analyses: Me, CV, Kruskal-Wallis ANOVA, Wilcoxon paired order test.

**Subjects**	**Number of sessions Participation time**	**Me CV**		
			**Measurement during the first session (pre)**	**Measurement during the last session (post)**
P1	5 1 month	78.00 12.033	66.00 25.296	75.00 13.036
P2	4 1 month	82.00 16.233		
P3	28 6 months	72.00 52.705		
P4	17 6 months	75.00 21.361		
P5	18 12 months	66.00 45.565		
P6	12 12 months	77.00 40.420		
		H (5, *N* = 84) = 2.912 *p* <0.713	*Z* = 8386 *p* <0.401	

*H, result of Kruskal-Wallis ANOVA factor analysis of the differences between the scores obtained by each participant during the entire intervention; Z, Wilcoxon paired-rank order test of the differences between the scores obtained in the pre- and post-test in the group of six participants; P, patient; Me, median values; CV, coefficients of variation*.

Kruskal-Wallis factor ANOVA analysis revealed no statistically significant differences (*p* < 0.713) between the scores obtained by each participant throughout the intervention. There were also no significant differences between the scores obtained in the pre- and post-intervention measures (*p* < 0.401). However, it should be noted that there was less variation in the scores in the post-measurement (CV = 13.036) compared to the pre-measurement (CV = 25.296), and some tendency for scores to be higher in the post-measurement (Me = 75.00) compared to the pre-measurement (Me = 66.00). This indicates greater homogeneity of results in the post-measurement.

## Discussion

The process of neurodegeneration consists in the gradual disappearance of brain tissue because of a lack of stimuli reaching the basal ganglia and the cerebral cortex (22, 23). It is, therefore, necessary that the patient is correctly diagnosed and undergoes proper rehabilitation, including neurorehabilitation. It aims to minimize the effects of damage to the CNS, including among others cognitive impairments (24, 25). The special role of such impairments is emphasized by Pachalska (26), who claims that cognitive and behavioral disorders lead to deeper severe impairments than do motor problems.

In previous studies on the cognitive functions in patients with damage to the CNS, researchers used traditional psychological and psychiatric testing instruments, most of which required verbal contact with the patient, and neuroimaging or dichotic methods, which were applied to assess patients' attention (13, 27–29). The use of the visual communication channel in the examination and practice of cognitive functions seems to be a perfect solution for patients who are unable to communicate verbally and have no motor ability to communicate their needs to their caregivers (30). In light of the presented results, we found that this communication channel, which has not been used so far in the traditional assessment of patients with damage to the CNS, has the potential for use as an alternative method of assessment among patients incapable of verbal and motor communication. We found out that patients diagnosed with UWS did in fact establish contact with the therapist and managed to perform cognitive tasks. This implies that, although it is quick and easy to use, the GCS (which is currently used in Poland) is not an effective tool for making the best clinical decisions. For this reason, it should be replaced by a more advanced tool, which is the Coma Recovery Scale-Revised (CRS-R) (31, 32).

Comprehensive stimulation of patients diagnosed with UWS is particularly important because, according to Gerald Edelman's theory of Neural Darwinism, early rehabilitation of cognitive functions contributes to the maintenance of as many neuronal connections in the brain as possible (33–36). The theoretical basis of the neuroplasticity of the brain suggests that the creation of new neuronal connections is affected by comprehensive stimulation of the nervous system (37–43), which can be regarded as the multimodal neural stimulation presented in a paper by Krucoff et al. (44). This article presents, among others, brain-machine interfaces whose task is to extract information from the nervous system and decode it to generate functional output. These steps are taken to bypass the damage occurring in the brain. A review of studies in the paper by these authors indicates that currently brain-machine interfaces are also thought to improve neural plasticity and enhance recovery. Therefore, we conclude that using eye-tracking can be one of such interfaces contributing to the improvement of patients' cognitive functions. Further, the use of the neuronal path responsible for sight is supposed to improve eyeball movements and thereby increase the effectiveness of exercises carried out with the eyes.

Eyeball movements are controlled by three cranial nerves (III, IV, and VI). The literature underlines the role of small muscle training because the movements of the eyeball influence the coordination of the suboccipital muscles, which, in turn, influence the coordination of the paraspinal muscles, translating into the overall motor activity of the human body (45, 46). The quality of eye movements is also influenced by the cerebellum and the basal nuclei. The cerebellum primarily regulates the speed and coordination of all eye movements, and a damage to the cerebellum can lead to the elimination of fixation movements. The basal nuclei are responsible for saccadic movements, the velocity of which is impaired when they are damaged (47). However, most of the patients after the damage to the CNS experience problems with movements of the eyeballs (fixation or saccadic eye movement) (48). This emphasizes the importance of using eyeball-controlled devices. They fulfill the task of training and stimulating the muscles responsible for eye movements. The patient's capability of eye fixation is an important sign of a return to functionality (49). The research on eye fixation was conducted by Trojano et al. (50) in three groups of subjects (9 in a MSC, 9 with UWS, and 11 subjects making up the control group). Eye fixation time did not differ significantly between the groups, whereas the proportion between keeping the eyes on the target and off the target was significantly different between the MCS and UWS groups.

Positive effects of work that relied on eye-tracking have been observed in patients at the intensive care unit (ICU) (51). The research was concerned with the effect of an eye-tracking device on the possibility of improving patients' communication and psychosocial condition. All the patients from the ICU were able to communicate their basic needs to their families and to the nursing personnel. The results suggested that the eye-tracking device improved their psychosocial condition and communication with the people around them. The eyeball movements were also applied in the research conducted by Kwiatkowska (52). The experiment concerned the writing and reading functions in people with decreased awareness and those who had awoken from a coma. The results revealed that in patients with decreased awareness, global reading ability was retained, particularly with regard to individual words and sentences. These results correspond with our own research, where patients with consciousness disorders succeeded in completing a significant proportion of tasks assessing language functions.

The importance of using alternative communication tools is also demonstrated by the results obtained by Poletti et al. (53). These researchers analyzed the possibilities for healthy people to perform psychological and cognitive tests using eye trackers. In the conclusion of their work, the authors proposed to perform a psychological assessment using eye trackers among people with verbal-motor limitations, when “traditional assessment instruments” were not suited to their dysfunctions. These results are confirmed by our own research, as patients were willing to cooperate when given the possibility to communicate using the right tool and contact with them was possible. Similar reasoning can also be found in a paper by Yu et al. (54). Moreover, the discrepancy between reactivity and preserved consciousness in patients, which results in the need to revise the diagnosis of patients with UWS is also pointed out by Laureys and Boly (55).

Based on the results of our study, it is not possible to unambiguously conclude that there was an improvement in the language functions in all participants. It is not fully clear, either, why the results sometimes decreased, as well as why the results have sometimes radically changed between sessions. Some participants in the study exhibited the above mentioned decrease in scores despite the same length of time in the study but different amounts of sessions. The more frequent visits by the therapist might be the way to explain why the scores decreased. It is likely that patients may have been influenced by weather conditions, lack of family visits, or mood changes resulting from the condition they were in. Above all, it should be mentioned that the level of consciousness in DOC patients is not constant but can fluctuate (9) and decrease on a certain day of the therapy, thus affecting their scores on language tasks. What certainly needs to be emphasized is that, in statistical analyses, less post-variation of the results and high scores were obtained across the group in both pre- and post-intervention measures. Additionally, we believe the patients' levels of communication abilities were not due to a spontaneous improvement from the first 3 months after the trauma, as all our participants were included in the project from the 4th month onward after the trauma. However, there seem to be other factors that are relevant to the will to communicate (or lack of it) that cannot be explained with the current state of knowledge.

## Limitations

A limitation in the present study might be the fact that the break time in the patients' work during the therapeutic sessions was not measured. This information could be helpful in analyzing the changes in the results. Another limitation is the irregular intervals between sessions and the low number of patients participating in the study. It is possible to provide regular neurorehabilitation, but cooperation with the patient depends on their neurophysiological condition on a given day. Future studies could, however, seek to include a higher number of patients in order to develop the diagnostic application of eye-tracking devices among UWS patients.

## Conclusions

The results of the study present changes that occurred in the language functions of patients, measured in consecutive sessions conducted at different time intervals.

The mean percentage of tasks completed was high, regardless of how long patients participated in the study, demonstrating the potential capabilities of patients with UWS. The results showed high intra-individual variability, which is typical in this group of patients. Despite the lack of demonstrated efficacy of rehabilitation sessions, the high percentage of tasks completed by patients allows for the consideration of eye tracking as an alternative method of neurorehabilitation and assessment for patients unable to communicate verbally and motorically. This may suggest that diagnoses of patients' states of consciousness may be more objective when an eye-tracking device is used to assess their potential.

In our study, the patients diagnosed with UWS were still able to perform tasks, suggesting they were not so much UWS patients, which they are diagnosed as, but MCS patients. This means that their diagnoses were incorrect due to the use of an incorrect diagnostic tool.

## Data Availability Statement

The raw data supporting the conclusions of this article will be made available by the authors, without undue reservation.

## Ethics Statement

The studies involving human participants were reviewed and approved by the Institutional Bioethical Committee at University School of Physical Education in Wroclaw (consent number: 29/2017). The patients/participants provided their written informed consent to participate in this study. Written informed consent was obtained from the individual(s) for the publication of any potentially identifiable images or data included in this article.

## Author Contributions

Material preparation, data collection, and analysis were performed by KK, AŻ, AK, GZ, and RO. The first draft of the manuscript was written by KK, AŻ, and GZ. AK, GZ, and RO contributed substantially to the interpretation of the results, provided critical feedback, and revised the manuscript. All authors contributed in review and editing of the manuscript and approved its final version. All authors contributed to the study conception and design.

## Conflict of Interest

The authors declare that the research was conducted in the absence of any commercial or financial relationships that could be construed as a potential conflict of interest.
